# The Inter-Regional Connectivity Within the Default Mode Network During the Attentional Processes of Internal Focus and External Focus: An fMRI Study of Continuous Finger Force Feedback

**DOI:** 10.3389/fpsyg.2019.02198

**Published:** 2019-09-26

**Authors:** Zhi-Wei Zhou, Xia-Qing Lan, Yan-Tong Fang, Yun Gong, Yu-Feng Zang, Hong Luo, Hang Zhang

**Affiliations:** ^1^Institute of Psychological Sciences, College of Education, Hangzhou Normal University, Hangzhou, China; ^2^Center for Cognition and Brain Disorders and the Affiliated Hospital, Hangzhou Normal University, Hangzhou, China; ^3^Zhejiang Key Laboratory for Research in Assessment of Cognitive Impairments, Hangzhou, China

**Keywords:** sustained attention, external focus/internal focus, fMRI, continuous finger force feedback, functional connectivity

## Abstract

Sustained attention involves two distinct processes, i.e., external focus and internal focus. Some recent neuroimaging studies employed the instruction of experimenters or the self-report from participants to generate the two attentional processes, and observed that the default mode network (DMN) was also responding to the external focus. These observations challenged the general view that the DMN accounts for the internally directed cognition, e.g., unfocused mind wandering, task independent-thoughts and internally focused events. Notably, the instruction or self-report may not effectively ensure the participants engage in the external focus/internal focus, and thus, the functional significance of the DMN for the externally focused process remains to be verified. In the present study, a new task paradigm, i.e., real/sham continuous feedback of finger force, was employed to generate the attentional process of external focus/internal focus, and the functional connectivity among the node regions of the DMN was further investigated in the two processes respectively. We found that two regions of the DMN, posterior cingulate cortex and left inferior parietal cortex/angular gyrus showed stronger inter-regional connectivity in the externally focused process than it in the internally focused process. Intriguingly, this functional connectivity was closely related to the behavioral performance in the process of external focus. These findings implicated that the functional significance of the DMN in sustained attention was more than responding to the internally directed cognition, and the task paradigm of continuous finger force feedback could benefit for the future studies on the externally focused/internally focused process of sustained attention.

## Introduction

Almost every task in the daily life requires human to maintain the attentional focus. Sustaining attention over prolonged periods of time is of great interest to cognitive psychologists. This is recognized by the fact that the sustained attention is critical for successful cognitive processing (Coull et al., 1996), and a number of psychiatric disorders, e.g., attention deficit hyperactivity disorder (Liu et al., 2015), schizophrenia (Burton et al., 2018), depression (Ye et al., 2018), etc., always show symptoms of deficits in sustained attention. The increasing number of evidences indicate that sustained attention could be dissociated into two attentional processes based on whether the attentional focus is external or internal (Chun et al., 2011; Fortenbaugh et al., 2017). The attentional process of external focus refers to external information of tasks, e.g., locations in space, the shape of targets, etc., while the process of internal focus refers to the internal experience of tasks, such as rules, decisions, responses, etc (Chun et al., 2011). Behavioral investigations suggested that the externally focused process could promote the performance in many sport items, e.g., volleyball, dart throwing and so forth (Wulf et al., 2002; Shafizadeh et al., 2013), and the internally focused process benefits the performance when the context was related to specific movement form rather than the performance outcomes (Poolton et al., 2006; Schücker et al., 2014). Of note, these intriguing findings came from investigations on quite different paradigms, such as probe-detection paradigm (Mansell et al., 2003), thought-probe paradigm (Scheibner et al., 2017) and so on. In these paradigms, the attentional processes of external focus and internal focus were mostly generated through the instruction of experimenters or self-reports from participants (Wulf et al., 2002; Scheibner et al., 2017). Actually, the instruction and self-report were mostly subjective, and may not effectively ensure the participants engage in the externally focused/internally focused process, and thus, this uncertainty potentially confounds the previous findings. Our research group has proposed a task paradigm, i.e., continuous finger force feedback (Dong et al., 2012). In this paradigm, the attentional process of internal focus/external focus could be generated through the real/sham feedback condition. In the real feedback condition, visual feedback of the finger force severed as the external focus for participants to maintain the finger force, and in contrast, participants, in the sham feedback condition, should maintain the finger force according to their sensory feeling and memory. This allows generating the externally focused and internally focused processes through similar tasks, and behavioral data for each process could be acquired respectively. With this paradigm, it was observed that participants controlled the finger force at the same level no matter they engage in the attentional process of external focus/internal focus, but the internal focus induced greater behavioral variation than the external focus (Dong et al., 2012). Nevertheless, the brain mechanism underlying these behavioral findings remains to be understood.

Functional magnetic resonance imagining (fMRI) studies informed us with the brain activity underlying sustained attention. The prominent role of a brain network, i.e., default mode network (DMN) was intensively discussed in these studies (Raichle et al., 2001; Damoiseaux et al., 2008; Uddin et al., 2010). The DMN consistently shows higher activity at rest compared to tasks requiring sustained attention (Whitfield-gabrieli and Ford, 2012; Danckert and Merrifield, 2016). Higher activity of the DMN was suggested to be related to mind wandering (Andrews-Hanna, 2012; Mittner et al., 2016) and task-independent thoughts (Mason et al., 2007; Korostil et al., 2016). Increased activity of the DMN was linearly linked to intensity of awareness of internal focus (Vanhaudenhuyse et al., 2011). These evidences support the functional role of the DMN in internally directed cognition. In contrast, recent studies showed the DMN may also engage in the attentional process of external focus (Bogler et al., 2017; Scheibner et al., 2017). Scheibner et al. (2017) employed the instructions of experimenters and the self-reports from participants to generate the external focus (sound) and the internal focus (breathing), and they observed that the meditation practice reduced the activity of the DMN in the both tests of external focus and internal focus (Scheibner et al., 2017). Bogler et al. (2017) found the activity in the DMN was higher when subjects had a relatively short reaction time in a vigilance task (Bogler et al., 2017). However, no results in the report of Scheibner et al. (2017) showed the relationship between the DMN activity and the behavioral performance. Bogler et al. (2017) did not verify their findings in the attentional process of internal focus. Therefore, whether the DMN engaged in the externally focused process remains to be further validated. It is worthy to note that the DMN consists of several node regions including ventral and medial prefrontal cortex (vmPFC), the posterior cingulate cortex (PCC) and the left/right inferior parietal lobe/angular gyrus (LIPC/AG and RIPC/AG) (Raichle et al., 2001; Broyd et al., 2009; Greicius et al., 2009). In our previous studies, these regions showed higher activity in the real feedback condition than it in the sham feedback condition when we performed voxel-based analyses with the measurements of amplitude of low frequency fluctuation and regional homogeneity (Dong et al., 2012; Zhang et al., 2015a). Since no correlation between behavioral performance and regional activity were observed, these investigations offered few evidences for understanding the functional role of the DMN in sustained attention. Actually, regions constitute DMN through the functional connectivity which was methodologically defined as the correlation between the time course of a particular brain region and other regions (Friston et al., 1993). This functional connectivity could also be observed among different brain networks (Wang et al., 2014; Breakspear, 2017). These inter-regional and inter-network interactions were believed as the fundamental support for many cognitive processes, e.g., emotional modulation, skill learning, etc. (Mahiko et al., 2015; Nusslock et al., 2019). In our previous studies, we have explored the inter-network interactions, and the connectivity among the DMN, the executive network and the left frontal-parietal network exhibited changes between the attentional processes of internal attention and external focus (Zhang et al., 2015b). However behavioral performance did not show any correlation with the connectivity among these brain networks. Thus, the inter-regional connectivity was further assessed here to clarify the functional role of DMN in the attentional process of external focus/internal focus.

The present study examined the connectivity among the node regions of DMN for verifying whether the DMN was also related to the attentional process of external focus. We hypothesized that if the DMN was related to the externally focused process, stronger connectivity could be identified in the process of external focus, and the connectivity among the node regions of the DMN should exhibit significant correlation with behavioral performance in this process. To test these hypotheses, fMRI data from our previous study were re-analyzed (Dong et al., 2012). The attentional processes of external focus and internal focus were established through the paradigm of continuous finger force feedback, and inter-regional connectivity within the DMN and their relations to behavior performances were assessed in each process separately.

## Materials and Methods

### Participants

Forty-three right-handed college students participated in the study (23 ± 3 years, range 19–25; 23 females). No participant had history of brain injury, neurological illness or psychiatric disorders. Five subjects were excluded for the malfunction of experimental equipment (three subjects, leakage from the air tube resulted in the negative value of finger force) or excessive head motion (two subjects, head motion was >2 mm translation or >2° rotation in any direction), and at last, data from 38 subjects (mean age, 22 ± 2 years; 19 females) were involved in the further analysis. All experiments conducted in this study were approved by the Institutional Review Board of National Key Laboratory of Cognitive Neuroscience, Beijing Normal University. All the subjects gave written informed consent before the experiment.

### Experimental Design

The current data were from our previous studies, and four papers have been published based on the data (Dong et al., 2012; Zhang et al., 2015a,b,c). Dong et al. (2012) proposed the finger force feedback paradigm and reported the behavioral data. Zhang et al. (2015a, c) were two methodological studies and provided the methodological framework for the exploration with this new paradigm. Zhang et al. (2015b) examined the functional connectivity among several brain networks, and no inter-regional functional connectivity within any one of the brain networks was analyzed (Zhang et al., 2015b). Each participant first underwent a scanning of resting state for adapting to the fMRI environment. Then, two sessions of external focus/internal focus were performed with the order counterbalanced across all participants. Each session lasts for 8 min, and the participants had a short practice period to get familiar with the related procedure before each session. In the session of external focus, the participants pinched a pressure sensor between the right index finger and thumb. This sensor is one module of an MRI-compatible physiological multi-channel analyzer (model MP150, BIOPAC Systems, Inc., Goleta, CA, United States). The sampling frequency was 250 Hz, and the pressure sensitivity was 0.01 cm H_2_O. The pressure was recorded by a sensor via an airtight tube, and the force of pressure was synchronously fed back to the participant on a projector as the external focus. At the same time, each participant was requested to continuously regulate the finger force and try to maintain it at 20 cm H_2_O according to the feedback. This target force was set in order to reduce the possibility of muscular fatigue for all subjects (van Duinen et al., 2007). In the session of internal focus, participants also maintained the finger force at 20 cm H_2_O, and they should maintain the finger force according to their sensory memory and experience from the practice period but not from the feedback. Participants also watched a sham feedback to keep the visual inputs consistent across different sessions, and this feedback was generated with the behavioral data of another participant in external focus session. Because the sham feedback of finger force could be easily detected, we have informed participants of this fact in advance and requested them to keep their own performance unaffected.

### Data Acquisition

Scanning was performed at the MRI Center of the Beijing Normal University using a 3.0-T Siemens whole-body MRI scanner. A single-shot T2^∗^-weighted, gradient-echo, EPI sequence was used for functional imaging acquisition with the following parameters: repetition time (TR)/echo time (TE) = 2000 ms/30 ms, flip angle = 90°, acquisition matrix = 64 × 64; field of view (FOV) = 200 mm × 200 mm and slice thickness/gap = 3.5/0.7 mm. Thirty-three axial slices parallel to the AC-PC line were obtained in an interleaved order to cover the entire cerebrum and cerebellum. Then a T1-weighted three-dimensional magnetization-prepared rapid gradient echo (MPRAGE) sequence was acquired [128 sagittal slices, slice thickness/gap = 1.33/0 mm, in-plane resolution = 256 × 192, TR/TE/inversion time (TI) = 2530/3.39/1100 ms, flip angle = 7°, FOV = 256 mm × 256 mm].

### Data Preprocessing

The preprocessing was carried out using the Data Processing Assistant for Resting-State fMRI (DPARSF) (Yan and Zang, 2010), which is based on the Statistical Parametric Mapping (SPM8)^1^ and Resting State fMRI Data Analysis Toolkit (REST) (Song et al., 2011)^2^. The first 10 time points were removed for signal stabilization and participant adaptation, and then, the images were corrected for the difference in slice acquisition timing and head motion, coregistered to the T1 structural image. The head motion parameter measured by Friston-24 model and signals from white matter (WM) and cerebrospinal fluid (CSF) were further regressed out as nuisance covariates, and the linear trends were removed from the time courses of the voxels in each image. Then, images were spatially normalized into the standard Montreal Neurological Institute (MNI) template (re-sampled into 3 mm × 3 mm × 3 mm) and smoothed with an 8 × 8 × 8 full-width-at-half-maximum (FWHM) Gaussian kernel.

### DMN Extraction With Independent Components Analysis

The preprocessed data from the external focus and internal focus sessions were combined into one single-group ICA analysis using the GIFT software^3^, and the optimal component number in the analysis was estimated to be 20 according to the minimum description length (MDL) criteria (Calhoun et al., 2002). Two-step PCA was used to reduce the dimensionality of data to 20. Next, the data were decomposed by ICA using the informax algorithm (Bell and Sejnowski, 1995). To ensure the stability of the decomposition, ICASSO (Himberg et al., 2004) with 10 ICA runs were used (Ge et al., 2019), and the most stable run was selected as the final result. Then, spatially independent components (ICs) were back reconstructed for each subject, and at last, 20 ICs and the related time courses of responses for each subject were acquired. These ICs were converted to z-maps, and one-sample *t*-test was further performed to determine the group spatial map of DMN for subjects in the session of external focus/internal focus respectively (*p* < 0.001, GRF correction).

### Functional Connectivity Among Regions of DMN

The functional connectivity was first analyzed based on the ICA results. Regions of the spatial map of DMN, including vmPFC, PCC, RIPC/AG and LIPC/AG were identified as the regions of interest (ROIs). A sphere with a 9-mm radius centered at the peak MNI coordinates of each ROI was defined as the seed region (external focus session, vmPFC: *x* = 6, *y* = 57, *z* = 15; PCC: *x* = 0, *y* = −51, *z* = 33; LIPC/AG: *x* = −42, *y* = −72, *z* = 36; RIPC/AG: *x* = 45, *y* = −63, *z* = 33; internal focus session, vmPFC: *x* = 0, *y* = 51, *z* = 15; PCC: *x* = −6, *y* = −60, *z* = 24; LIPC/AG: *x* = −39, *y* = −75, *z* = 36; RIPC/AG: *x* = 42, *y* = −63, *z* = 30). Then, the preprocessed image data were filtered to 0.01–0.08 Hz, and the mean time course of each seed region was extracted. Functional connectivity between each pair of two seed regions was calculated through Pearson correlation coefficient. The Fisher *z*-transformed correlation coefficients identified as the DMN connectivity were compared between the two sessions using paired *t*-test, and all of the tested results underwent the multiple comparison correction [false discovery rate (FDR) correction *q* < 0.05].

These analyses were validated by using the ROIs with a different radius (6 mm). According to the above statistical results, the DMN connectivity showing significant difference between the sessions of external focus and internal focus was identified, and the identified functional connectivity was involved in the further analysis.

### Correlation Between Functional Connectivity and Behavior

The behavioral data of the external focus and internal focus sessions have been analyzed and illustrated in our previous investigation (Dong et al., 2012). Three measurements of intra-individual behavior were calculated, and the measurements include intra-individual mean finger force (II_Mean, the mean value of finger force across a whole session), intra-individual standard deviation (II_SD, the SD of the individual pinch force across a whole session) and intra-individual variation coefficient (II_CV calculated as SD/mean value of the individual pinch force across a whole session). Then, the correlation between the identified functional connectivity and each of the behavioral measurements was calculated in the external focus/internal focus session respectively.

All the results were further validated by reproducing the functional connectivity analysis based on a public DMN spatial template (Greicius et al., 2004). vmPFC, PCC, RIPC/AG, and LIPC/AG in the template were defined as the seed region. Then, the mean time course of each seed region was extracted based on the filtered image data, and functional connectivity between each pair of two seed regions was calculated with Pearson correlation coefficient. The Fisher *z*-transformed correlation coefficients were further compared between the attentional processes of external focus and internal focus using paired *t-*test, and all of the tested results were further corrected for multiple comparison (FDR correction, *q* < 0.05).

## Results

### The DMN for the Attentional Processes of Internal Focus and External Focus

**FIGURE 1:**
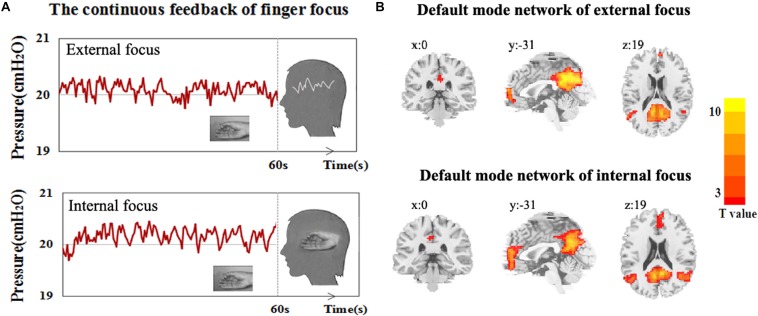
The experiment procedure and DMN spatial map. **(A)** The experimental diagram of the attentional process of external focus/internal focus generated by continuous finger force feedback task, and **(B)** DMN spatial map of external focus/internal focus process identified with ICA.

The attentional process of external focus/internal focus was generated with real/sham feedback condition (Figure 1A). The DMN spatial maps, identified in the attentional processes of external focus and internal focus were shown in Figure 1B. In both processes, the DMN spatial map involves regions of ventral and medial prefrontal cortex (vmPFC), posterior cingulated cortex (PCC)/precuneus and left and right inferior parietal cortex/angular gyrus (LIPC/AG and RIPC/AG) and the peak MNI coordinates of each region was showed in Table 1.

**TABLE 1:** Regions significantly recruited within DMN spatial map during the attentional process of external focus/internal focus.

**Involved brain regions for DMN**	**L/R**	**BA**	**Peak MNI coordinates**			
			***x***	***y***	***z***	***t*_max_**
**The attentional process of external focus**						
vmPFC	R	10	6	57	15	6.46
PCC	−	23	0	−51	33	8.28
LIPC/AG	L	19	−42	−72	36	7.32
RIPC/AG	R	39	45	−63	33	7.59
**The attentional process of internal focus**						
vmPFC	–	32	0	51	15	6.28
PCC	L	23	−6	−60	24	8.30
LIPC/AG	L	19	−39	−75	36	9.57
RIPC/AG	R	39	42	−63	30	7.55

*The statistical threshold was set at *p* < 0.001, GRF corrected.*

### Inter-Regional Connectivity Within the DMN

**FIGURE 2:**
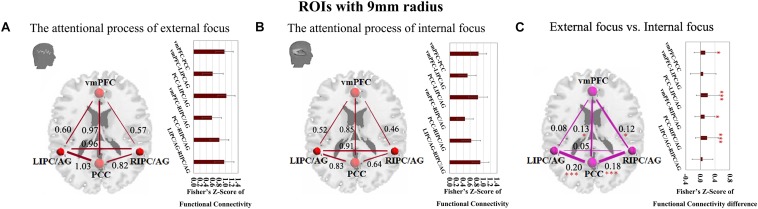
The inter-regional connectivity between each pair of the regions within the DMN during the attentional process of external focus/internal focus. **(A)** The visualization of significant connectivity during the attentional process of external focus. **(B)** The visualization of significant connectivity during the attentional process of internal focus. In **(A,B)**, line width indicates the relative value of the connectivity. **(C)** The difference of the connectivity between the attentional processes of external focus and internal focus, and the line width indicates the relative value of the connectivity difference between the two processes. ^∗^Indicates the significant difference, *p* < 0.05; ^∗∗∗^indicates the significant difference, *p* < 0.001, FDR corrected (Sphere radius = 9 mm).

The four critical regions of the DMN showed six pairs of inter-regional connectivity with each other (Figures 2A,B), and the inter-regional connectivity within the DMN was significant in both external focus and internal focus processes (each *t* > 11.40, *p* < 0.0001). Four pairs of the connectivity exhibited significant differences between the attentional processes of external focus and internal focus, including vmPFC-PCC, PCC-LIPC/AG, vmPFC-RIPC/AG and PCC-RIPC/AG (see details in Figure 2C and Table 2).

**TABLE 2:** The inter-regional connectivity within DMN of the attentional process of external focus/internal focus, and the comparison results of the inter-regional connectivity between the processes of external focus and internal focus.

**Conditions**	**The attentional process of external focus**	**The attentional process of internal focus**	**External focus vs. internal focus**	
**DMN connectivity**	***Fisher’s Z-score, Mean* ± *SD***	***Fisher’s Z-score, Mean* ± *SD***	***t(37)***	***p***
vmPFC-PCC	0.97 ± 0.27	0.85 ± 0.23	2.69	0.01^∗^
vmPFC-LIPC/AG	0.60 ± 0.33	0.52 ± 0.26	1.41	0.17
PCC-LIPC/AG	1.03 ± 0.27	0.83 ± 0.27	3.97	0.0003^∗∗∗^
vmPFC-RIPC/AG	0.57 ± 0.31	0.46 ± 0.24	2.68	0.01^∗^
PCC-RIPC/AG	0.82 ± 0.30	0.64 ± 0.27	3.61	0.0009^∗∗∗^
LIPC/AG-RIPC/AG	0.96 ± 0.30	0.91 ± 0.25	1.17	0.25

*^∗^Indicates the significant difference, *p* < 0.05; ^∗∗∗^indicates the significant difference, *p* < 0.001, FDR corrected (Sphere radius = 9 mm).*

Since we have performed four studies based on the same data, thus, we further performed the multiple comparisons (taking into account previous correlations that we have done). Twenty comparisons between the two different attentional processes have been administrated. Using Bonferroni correction across all comparisons, the significant level for the comparisons in the current study is *p* < 0.0025 (0.05/20). Thus, the stronger connectivity of PCC-LIPC/AG and PCC-RIPC/AG for the externally focused process could withstand this multiple comparison correction (each *p* < 0.001).

**FIGURE 3:**
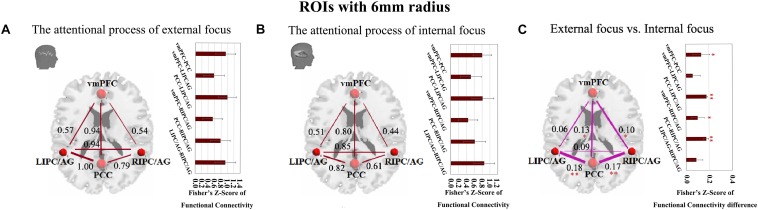
The inter-regional connectivity between each pair of the regions within DMN during the attentional process of external focus/internal focus. **(A)** The visualization of significant connectivity during the attentional process of external focus. **(B)** The visualization of significant connectivity during the attentional process of internal focus. In **(A,B)**, line width indicates the relative value of the connectivity. **(C)** The difference of the connectivity between the attentional processes of external focus and internal focus, and the line width indicates the relative value of the connectivity difference between the two processes. ^∗^Indicates the significant difference, *p* < 0.05; ^∗∗^indicates the significant difference, *p* < 0.005, FDR corrected. (Sphere radius = 6 mm).

Moreover, we further validated these results using the ROIs with a different radius (6 mm). The significant differences between the attentional processes of external focus and internal focus were identified in the functional connectivity of vmPFC-PCC, PCC-LIPC/AG, vmPFC-RIPC/AG and PCC-RIPC/AG (Figure 3 and Table 3).

**TABLE 3:** The inter-regional connectivity within the DMN of the externally and internally focused processes, and the comparison results of the inter-regional connectivity between the internally and externally focused processes.

**Conditions**	**The attentional process of external focus**	**The attentional process of internal focus**	**External focus vs. Internal focus**	
**Connectivity**	***Mean* ± *SD fisher’s Z-score***	***Mean* ± *SD fisher’s Z-score***	***t(37)***	***p***
vmPFC-PCC	0.94 ± 0.31	0.80 ± 0.23	2.55	0.02^∗^
vmPFC-LIPC/AG	0.57 ± 0.33	0.51 ± 0.27	1.02	0.31
PCC-LIPC/AG	1.00 ± 0.27	0.82 ± 0.28	3.31	0.002^∗∗^
vmPFC-RIPC/AG	0.54 ± 0.31	0.44 ± 0.25	2.41	0.02^∗^
PCC-RIPC/AG	0.79 ± 0.30	0.61 ± 0.28	3.34	0.002^∗∗^
LIPC/AG-RIPC/AG	0.94 ± 0.32	0.85 ± 0.26	1.84	0.07

*^∗^Indicates the significant difference, *p* < 0.05; ^∗∗^indicates the significant difference, *p* < 0.005, FDR corrected (Sphere radius = 6 mm).*

### Correlation Between Inter-Regional Connectivity Within the DMN and Behavioral Measurements

Behavioral data from the external focus and internal focus sessions have been analyzed and illustrated in our previous investigation (Dong et al., 2012). Paired *t*-tests showed that there was no significant difference in the II_Mean between the attentional processes of external focus and internal focus. II_Mean across all subjects was 19.97 ± 0.05 cm H_2_O for the external focus process and 19.72 ± 5.40 cm H_2_O for the internal focus process (*t* = 0.29, *p* > 0.05). However, the II_SD and II_CV in the internal focus process were markedly higher than it in the external focus process. II_SD across all subjects was 0.17 ± 0.07 cm H_2_O for the external focus process and 2.85 ± 1.34 cm H_2_O for the internal focus process (*t* = 12.46, *p* < 0.0001). II_CV across all subjects was 0.01 ± 0.003 cm H_2_O for the real feedback and 0.17 ± 0.11 cm H_2_O for the sham feedback (*t* = 8.64, *p* < 0.0001).

**FIGURE 4:**
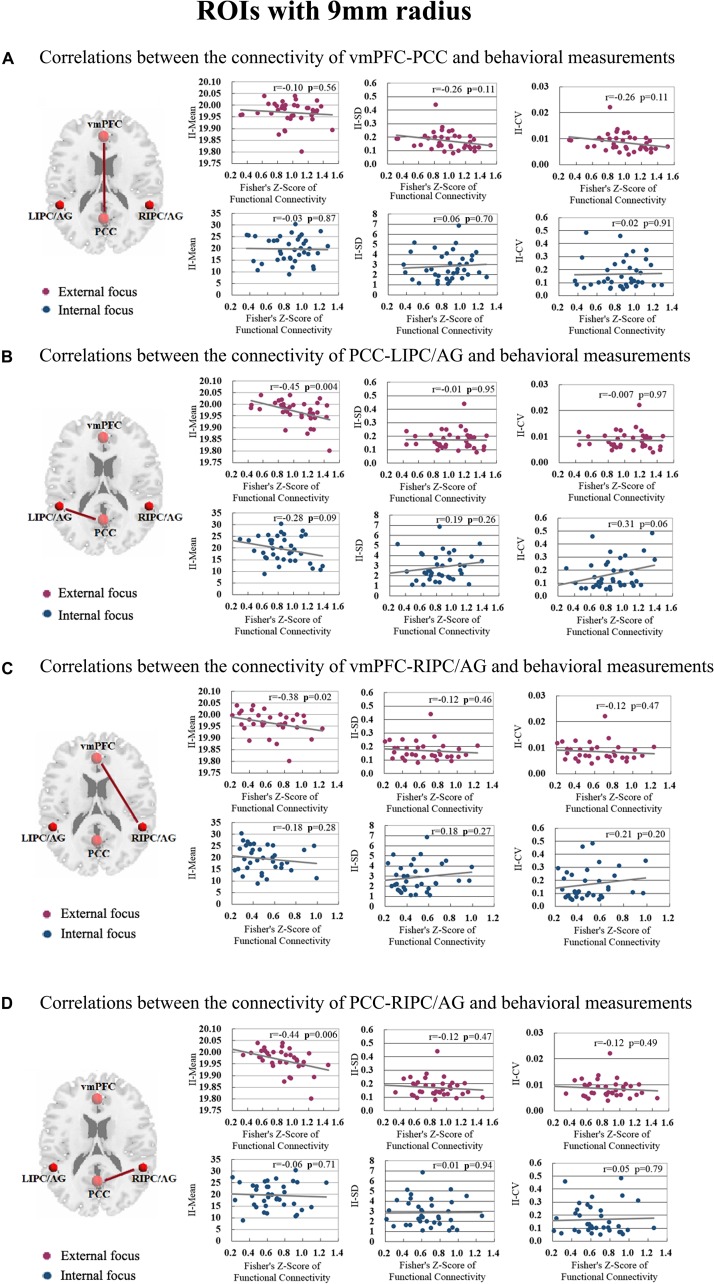
The correlations between the behavioral measurements, i.e., II_Mean, II_SD and II_CV and the functional connectivity of **(A)** vmPFC-PCC, **(B)** PCC-LIPC/AG, **(C)** vmPFC-RIPC/AG, and **(D)** PCC-RIPC in the attentional process of external focus/internal focus. (Sphere radius = 9 mm).

**FIGURE 5:**
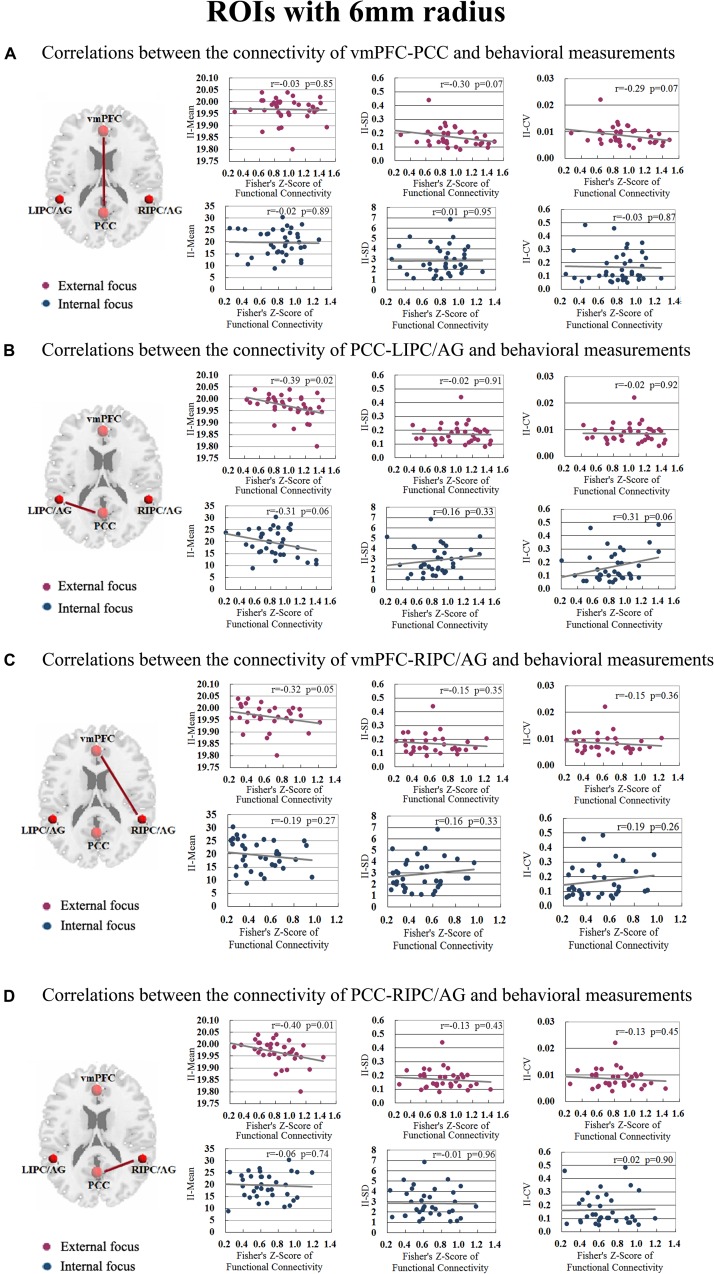
The correlations between the behavioral measurements, i.e., II_Mean, II_SD and II_CV and the functional connectivity of **(A)** vmPFC-PCC, **(B)** PCC-LIPC/AG, **(C)** vmPFC-RIPC/AG, and **(D)** PCC-RIPC in the attentional process of external focus/internal focus (Sphere radius = 6 mm).

The correlation between significant functional connectivity within DMN spatial map and behavioral measurement was showed in Figure 4. There was a significant correlation between the functional connectivity of PCC-LIPC/AG and the II-Mean of finger force in external focus process not internal focus process, and there was no significant difference between the functional connectivity of PCC-LIPC/AG and the II_SD or II_CV of finger force (Table 4). Moreover, these results were validated using the ROIs with a different radius (6 mm) (see details in Figure 5 and Table 5).

**TABLE 4:** The relationship between the inter-regional connectivity within DMN and behavioral measurements for the attentional process of external focus/internal focus respectively.

**Behavior**	**The attentional process of external focus**						**The attentional process of internal focus**					
**Connectivity**	**II_Mean**		**II_SD**		**II_CV**		**II_Mean**		**II_SD**		**II_CV**	
	***r***	***p***	***r***	***p***	***r***	***p***	***r***	***p***	***r***	***p***	***r***	***p***
**DMN spatial map identified by ICA**												
vmPFC-PCC	−0.10	0.56	−0.26	0.11	−0.26	0.11	−0.03	0.87	0.06	0.70	0.02	0.91
PCC-LIPC/AG	−0.45	0.004^∗∗^	−0.01	0.95	−0.007	0.97	−0.28	0.09	0.19	0.26	0.31	0.06
vmPFC-RIPC/AG	−0.38	0.02^∗^	−0.12	0.46	−0.12	0.47	−0.18	0.28	0.18	0.27	0.21	0.20
PCC-RIPC/AG	−0.44	0.006^∗^	−0.12	0.47	−0.12	0.48	−0.06	0.71	0.01	0.94	0.05	0.79
**DMN spatial template**												
PCC-LIPC/AG	−0.50	0.001^∗∗^	−0.05	0.77	−0.05	0.79	−0.1	0.55	0.03	0.86	0.08	0.61

*^∗^Indicates the significant difference, *p* < 0.05; ^∗∗^indicates the significant difference, *p* < 0.005 (Sphere radius = 9 mm).*

**TABLE 5:** The relationship between the inter-regional connectivity within DMN and behavioral measurements for the attentional process of external focus/internal focus respectively.

**Behavior**	**The attentional process of external focus**						**The attentional process of internal focus**					
**Connectivity**	**II_Mean**		**II_SD**		**II_CV**		**II_Mean**		**II_SD**		**II_CV**	
	***r***	***p***	***r***	***p***	***r***	***p***	***r***	***p***	***r***	***p***	***r***	***p***
**DMN spatial map identified by ICA**												
vmPFC-PCC	−0.03	0.85	−0.30	0.07	−0.29	0.07	−0.02	0.89	0.01	0.95	−0.03	0.87
PCC-LIPC/AG	−0.39	0.02^∗^	−0.02	0.91	−0.02	0.92	−0.31	0.06	0.16	0.33	0.31	0.06
vmPFC-RIPC/AG	−0.32	0.05^∗^	−0.15	0.35	−0.15	0.36	−0.19	0.27	0.16	0.33	0.19	0.26
PCC-RIPC/AG	−0.40	0.01^∗^	−0.13	0.43	−0.13	0.45	−0.06	0.74	−0.009	0.96	0.02	0.90

*^∗^Indicates the significant difference, *p* < 0.05 (Sphere radius = 6 mm).*

Since we have performed four studies based on the same data, thus, we further performed the multiple comparisons (taking into account previous correlations that we have done). Hundred and one correlations between the fMRI data and behavioral data have been assessed. Using Bonferroni correction across all correlations, no correlation results could withstand this multiple comparison correction (Bonferroni correction across all correlations, *p* < 0.0005).

**FIGURE 6:**
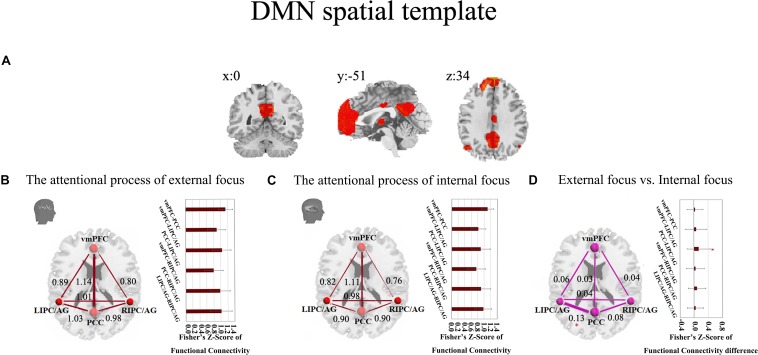
The inter-regional connectivity between each pair of the regions within DMN during the attentional processes of external/internal focus. **(A)** The public DMN spatial template. **(B)** The visualization of significant connectivity during the attentional process of external focus. **(C)** The visualization of significant connectivity during the attentional process of internal focus. In **(B,C)**, line width indicates the relative value of the connectivity. **(D)** The difference of the connectivity between the attentional processes of external focus and internal focus and the line width indicates the relative value of the connectivity difference between the two processes. ^∗^Indicates the significant difference, *p* < 0.05, FDR corrected.

These results were further validated with the DMN spatial template (Figure 6A). The six pairs of functional connectivity for the attentional processes of external focus and internal focus were show in Figures 6B,C, and only the functional connectivity of PCC-LIPC/AG reserved the significant differences between the attentional processes of external focus and internal focus (see details in Figure 6D and Table 6).

**TABLE 6:** The inter-regional connectivity within DMN and the difference of the functional connectivity between the attentional processes of external focus and internal focus.

**Conditions**	**The attentional process of external focus**	**The attentional process of internal focus**	**External focus vs. internal focus**	
**DMN Connectivity**	***Fisher’s Z-score, Mean* ± *SD***	***Fisher’s Z-score, Mean* ± *SD***	***t(37)***	***p***
vmPFC-PCC	1.14 ± 0.20	1.11 ± 0.17	0.92	0.37
vmPFC-LIPC/AG	0.89 ± 0.26	0.82 ± 0.23	1.26	0.22
PCC-LIPC/AG	1.03 ± 0.26	0.90 ± 0.29	2.52	0.01^∗^
vmPFC-RIPC/AG	0.80 ± 0.27	0.76 ± 0.28	0.95	0.35
PCC-RIPC/AG	0.98 ± 0.29	0.90 ± 0.31	2.01	0.05
LIPC/AG-RIPC/AG	1.01 ± 0.31	0.98 ± 0.26	0.97	0.34

*^∗^Indicates the significant difference, *p* < 0.05, FDR corrected.*

**FIGURE 7:**
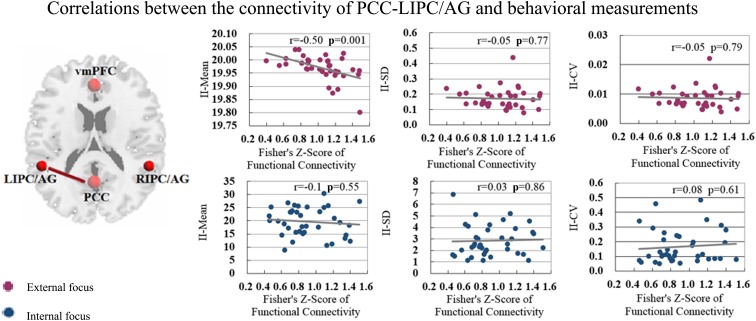
The correlations between the behavioral measurements i.e., II_Mean, II_SD and II_CV and the functional connectivity of PCC-LIPC/AG in the attentional process of external focus/internal focus.

The functional connectivity of PCC-LIPC/AG also showed significant correlation with the behavioral measurement of II-Mean in the attentional process of external focus (Figure 7 and Table 4).

## Discussion

The present study explored the functional significance of the DMN in sustained attention. The two processes were generated through a new paradigm, i.e., continuous feedback of finger force, and the functional connectivity among the node regions of the DMN was assessed in each process respectively. Two intriguing results were obtained: (1) the functional connectivity of PCC-LIPC/AG was significant stronger in the externally focused process than it in internally focused process, and this difference was reproduced in the validation analyses with different ROI radius and the public DMN spatial template; (2) the functional connectivity of PCC-LIPC was correlated with the behavioral measurement, II_Mean in the externally focused process. These findings potentially offer new insights into the functional significance of the DMN in the attentional processes of internal focus and external focus.

Since Chun et al. (2011) dissociated the sustained attention into the two attentional processes of internal focus and external focus (Chun et al., 2011), many studies attempt to identify the difference of behavioral performance and brain mechanism between the two attentional processes (Poolton et al., 2006; Stawarczyk et al., 2011; Shafizadeh et al., 2013; Schücker et al., 2014). Instructions and self-reports were the strategies mostly employed in these investigations to generate the attentional process of external focus/internal focus (Wulf et al., 2002; Ruocco and Direkoglu, 2013; Scheibner et al., 2017). However, these strategies could not be assessed by the objective behavioral measurements, thus it is difficulty to ensure subjects engage in these required processes. The paradigm of continuous feedback of finger force was employed in the present study, and controlling finger force by feedback and controlling finger force by the sensory memory were used to generate the external focus/internal focus. The finger force as the behavioral performance was recorded, and this paradigm potentially provide objective assessments for future studies on the attentional process of internal focus/external focus.

As hypothesized, we observed stronger functional connectivity within DMN in the attentional process of external focus. Previous studies suggested the involvement of the DMN in the internally directed cognition, e.g., mind wandering, task independent thoughts etc. (Jang et al., 2011; Benedek et al., 2016; Mittner et al., 2016). Here, the external focus increased the inter-regional connectivity of PCC-LIPC/AG as compared with the internal focus, and this result was still reserved in the validation analysis. Both PCC and LIPC were suggested to be related to working memory and information integrating (Ye and Zhou, 2009; Huang et al., 2018). In the current study, the functional connectivity of PCC-LIPC showed significant correlation with the behavioral measurement, II_Mean in the externally focused process. This attentional process requires processing and integrating the feedback information. Thus, PCC-LIPC within DMN may directly engage in the process of external focus, and probably regulated the behavioral performance related to the external focus. The dominant proposal argues that the DMN is mainly responsible for mind wandering, task independent thoughts (Andrews-Hanna, 2012; Seli et al., 2016; Scheibner et al., 2017; Bocharov et al., 2018). Thus, the functional role of DMN in sustained attention may be more than responding to the internally directed cognition.

Several limitations exist in current study. First, we observed that the stronger inter-regional connectivity of the DMN exhibited correlations with the behavioral measurement, II_Mean of the finger force in the externally focused process. However, these correlation results could not withstand the multiple comparison correction (each *p* < 0.01), if we took our previous studies based on the same data into account (Bonferroni correction across all correlations of the previous studies, *p* < 0.0005), thus the relationship between the inter-regional correlation and the behavioral performance in the process of external focus should be further verified. Second, The behavioral measurements, II_SD and II_CV were more meaningful for the assessment of the fluctuation of sustained attention (Liu et al., 2017). Whether the fluctuation of sustained attention was associated with the DMN remains to be understood. Third, for the internal focus, the relationship between the brain activity and behavioral performance in the internally focused process requires to be established in future studies, and we believed this is a critical issue for understanding of the brain mechanism underlying sustained attention.

## Conclusion

The present study explored the functional significance of the DMN in the attentional processes of external focus and internal focus. The external focus could increase the inter-regional connectivity, PCC-LIPC/AG of the DMN, and this connectivity within the DMN was possible the reason of regulating the behavioral performance in the externally focused process; These findings offered new evidences to support the engagement of the DMN in the attentional process of external focus. Thus, the functional significance of the DMN was more than the internally directed cognition, and the continuous feedback of finger force, as an objective assessing paradigm for sustained attention with external focus and internal focus deserves more concerns in future studies.

## Data Availability Statement

The datasets analyzed in this manuscript are not publicly available. Requests to access the datasets should be directed to kevinhangbnu@foxmail.com.

## Ethics Statement

The studies involving human participants were reviewed and approved by the Institutional Review Board of National Key Laboratory of Cognitive Neuroscience, Beijing Normal University. The patients/participants provided their written informed consent to participate in this study. Written informed consent was obtained from the individual(s) for the publication of any potentially identifiable images or data included in this article.

## Author Contributions

HZ and HL conceived and designed the experiment. Z-WZ and X-QL performed the data analysis. HL, Y-FZ, Y-TF, and YG provided advice on the analysis and interpretation of the results. Z-WZ, X-QL, and HZ wrote the manuscript.

## Conflict of Interest

The authors declare that the research was conducted in the absence of any commercial or financial relationships that could be construed as a potential conflict of interest.

## Footnotes

1 http://www.fil.ion.ucl.ac.uk/spm

2 http://www.restfmri.net

3 http://icatb.sourceforge.net
